# Definitive Chemoradiation With Dose Escalation for Locally Advanced Gastric Cancer: Case Studies

**DOI:** 10.7759/cureus.11040

**Published:** 2020-10-19

**Authors:** Michelle Anthony, Uma Goyal

**Affiliations:** 1 Radiation Oncology, University of Arizona College of Medicine, Tucson, USA; 2 Radiation Oncology, University of Arizona Cancer Center, Tucson, USA

**Keywords:** gastric cancer, definitive chemoradiation

## Abstract

Gastric cancer is a prevalent cancer with a predilection for Asian ethnicity and male patients. In early and locally advanced gastric cancer, without significant comorbidities, surgery is a part of the treatment management of this cancer. However, with concurrent comorbidities, surgery may not be recommended and alternative treatment options such as palliative chemotherapy and/or radiation and definitive chemoradiation can be considered to reduce morbidity. We present three cases of gastric cancer where definitive chemoradiation with dose escalation was utilized due to underlying comorbidities and poor performance status. The treatment was generally well tolerated by patients and resulted in substantial reduction in gastric mass size in two patients with median overall survival of 10 months.

## Introduction

Globally, there is a high prevalence of gastric cancer with more than 1 million cases being diagnosed in 2018. Worldwide, gastric cancer currently ranks as the fifth most common cancer diagnosis and third in cancer-related deaths. The incidence of gastric cancer is highest among males and those from eastern Asian countries [1]. The majority of gastric cancers are histologically adenocarcinoma, including intestinal and diffuse subtypes [2]. Potential risk factors for gastric cancer include alcohol consumption, tobacco smoking, and *Helicobacter pylori* [1-2].

Early gastric cancer patients generally have excellent prognosis [3]. However, the majority of patients have advanced gastric cancer at the time of presentation and a subset of these patients are unable to undergo definitive surgery due to underlying comorbidities [3-4]. Definitive chemoradiation has been proposed as an alternative to surgery in gastric cancer patients with comorbidities [3-4].

There is a paucity of studies assessing the effectiveness of definitive chemoradiation with dose escalation as a therapeutic option for unresectable gastric cancer in patients with poor performance status. The aim of this case series is to present three poor-performance status patients with unresectable locally advanced gastric adenocarcinoma that underwent definitive chemoradiation.

## Case presentation

Case one presentation 

A 63-year-old African American male with past medical history significant for hypertension presented with a two- to three-week history of progressively worsening abdominal pain, nausea, and vomiting. Patient endorsed dark stools a few weeks prior and 13-pound unintentional weight loss over a period of two months. His physical exam was significant for generalized muscle wasting and lab results showed anemia. He denied any tobacco, alcohol, or illicit drug use. Patient subsequently underwent abdominal CT followed by MRI, which revealed gastric outlet obstruction due to a 5.3 cm antropyloric mass with infiltration of the proximal duodenum, pancreatic head, and superior mesenteric vein. Subsequent biopsy confirmed gastric adenocarcinoma and staging workup showed a cT4bN0M0 gastric cancer. The patient was assessed by surgical oncology and was considered a poor surgical candidate because of invasion of the underlying superior mesenteric vein by the gastric mass. He was referred to radiation oncology and medical oncology. After discussion with the patient, a decision was made to pursue definitive treatment with chemoradiation.

He was planned for radiation therapy with a dose of 45 Gy in 25 fractions (fx) to the gastric mass and regional lymph nodes with a sequential boost to a total dose of 59.4 Gy in 33 fx to the gross disease using intensity modulated radiation therapy (IMRT) and daily image guidance (IGRT). The patient was asked to be nothing by mouth (NPO) two hours prior to radiation and also received concurrent capecitabine. For most of his treatment course, he tolerated the treatment well but did have tongue mucositis from chemotherapy. He began noticing dysphagia, occasional epigastric burning pain, and weight loss towards the end of his treatment course. As he approached the final three fractions of his boost radiation, due to nausea, vomiting and weight loss, the patient ended his treatment early, receiving a total dose of 54 Gy in 30 fx. 

**Figure 1:**
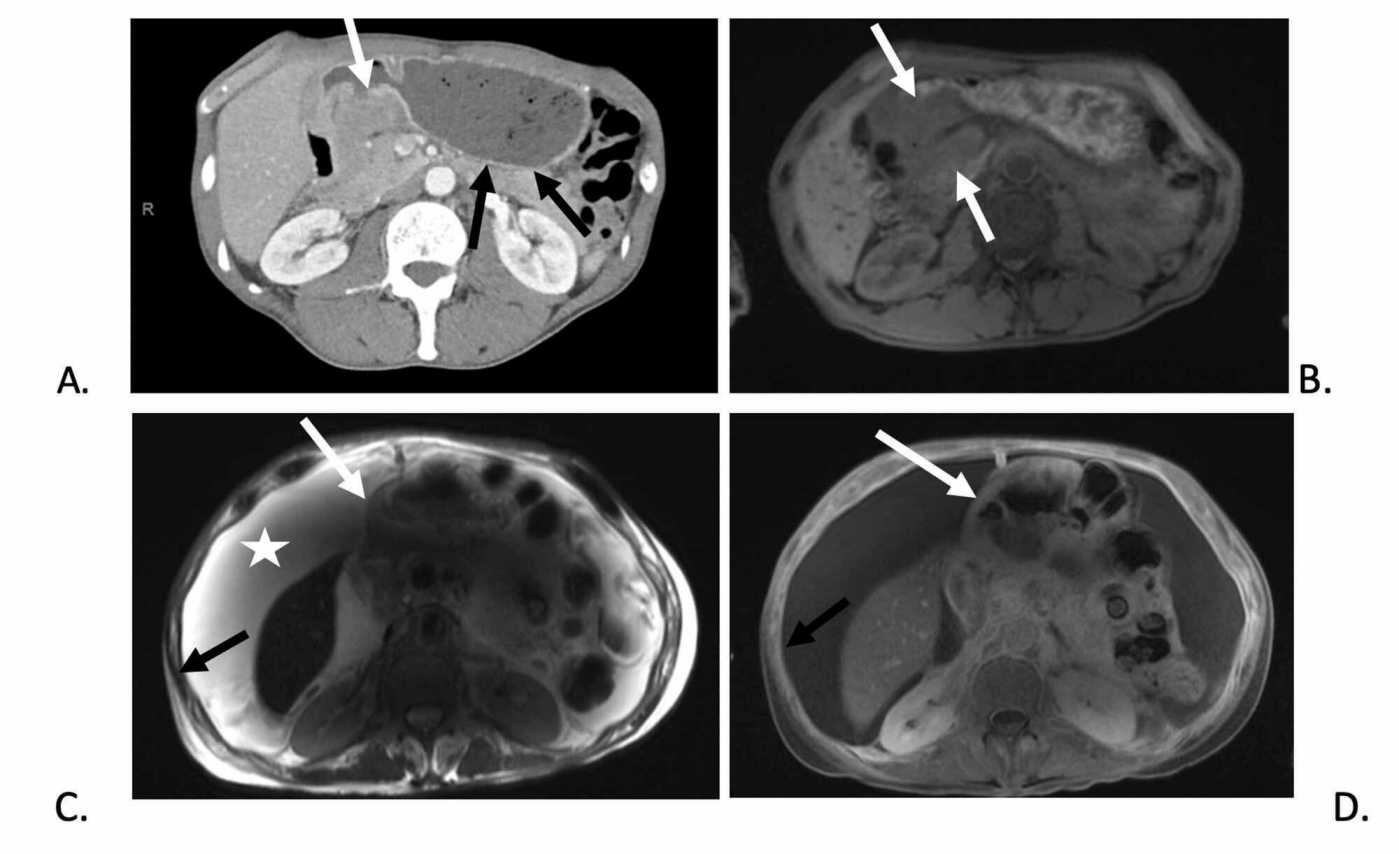
Case 1 imaging showing pyloric mass resulting in gastric outlet obstruction at presentation and treatment response of the gastric mass. (A) Contrast-enhanced axial CT and (B) pre-contrast T1-weighted axial MRI image shows a large gastric peripyloric soft tissue mass (white arrows) resulting in gastric outlet obstruction (black arrows). (C) T2-weighted axial MRI image and (D) post-contrast T1-weighted axial MRI image demonstrates marked improvement in size of the peripyloric mass (white arrows). However, patient did develop ascites (star) and peritoneal thickening (black arrows) which was reported to be reactive versus peritoneal carcinomatosis.

One day after his final radiation treatment, a CT abdomen was done due to ongoing symptoms and showed a favorable response to treatment as evidenced by reduction in size of the antropyloric mass. A three-month follow-up MRI of the abdomen showed continued excellent response to chemoradiation without significant residual tumor burden of the primary gastric antropyloric mass. However, the superior mesenteric vein occlusion was still present and extensive large volume ascites and peritoneal enhancement/thickening was also noted (Figure 1). Unfortunately, eight months later due to worsening of abdominal pain, nausea, and vomiting, the patient decided to enter into hospice. His primary tumor remained controlled, however, he likely died of distant progression.

Case two presentation 

A 69-year-old female with a history of stroke presented with abdominal pain, constipation, fecal incontinence, nausea, and vomiting. She denied any tobacco, alcohol, or illicit drug use. She underwent esophagogastroduodenoscopy, which found an ulcerated lesion in the gastric antropyloric region causing gastric outlet obstruction. Subsequent biopsy revealed gastric adenocarcinoma.

Staging MRI of the abdomen demonstrated two small masses (2.2x1.8 cm and 1.9x1.8cm) in the pancreatico-duodenal groove with resultant mild intrahepatic and extrahepatic biliary ductal dilation. An endoscopic ultrasound/endoscopic retrograde cholangiopancreatography was performed, which showed a focal distal biliary stricture with malignant appearance. A metal stent was placed in the common bile duct to relieve biliary obstruction.

She was referred to medical oncology and radiation oncology for further treatment of her cT4bN1M0 gastric adenocarcinoma. Since the patient was found to have bleeding of the gastric mass, her radiation was started urgently and she received 20 Gy in 10 fx to the bleeding gastric mass and regional lymph nodes, which resulted in the eventual cessation of bleeding. Further staging work-up showed no evidence of metastasis and she thus proceeded with definitive chemoradiation. The patient was asked to be NPO two hours prior to radiation. She received 27 Gy in 15 fx to the gastric mass and regional lymph nodes followed by 18 Gy in 10 fx boost to the gross disease. Concurrent treatment with capecitabine was given during radiation which was delivered with IMRT and daily IGRT. The total cumulative dose was 65 Gy in 35 fx. 

She tolerated her treatment course well, but had some underlying intermittent abdominal pain, dizziness, nausea, diarrhea, fatigue, dysphagia, and weight loss which was managed with medical management. A CT scan performed one-month post chemoradiation therapy could not visualize the known gastric cancer but did show persistent indeterminate soft tissue in the pancreatico-duodenal groove. A three-month follow-up MRI revealed continued excellent response to therapy without evidence of a distinct gastric lesion or metastatic disease. Unfortunately, a six-month positron emission tomography (PET)/CT scan showed progression with new metastatic mediastinal lymphadenopathy. She subsequently passed away three months later after suffering a myocardial infarction that was unrelated to her gastric cancer. 

Case three presentation 

A 80-year-old male with heart failure, coronary artery disease requiring stent placement, and previously diagnosed bladder cancer underwent esophagogastroduodenoscopy for iron deficiency anemia, which revealed a gastric mass. Subsequent biopsy of gastric mass identified a poorly differentiated gastric adenocarcinoma cancer with signet ring cells. An endoscopic ultrasound showed the gastric mass invading the muscularis propria and staging workup illustrated cT2N0M0 gastric cancer. There was mild stomach dilation on imaging but no measurable mass was seen. He was deemed a poor surgical candidate due to comorbidities and bleeding risk from anticoagulation therapy for his coronary artery stent placement. 

The patient was asked to be NPO two hours prior to radiation. The patient underwent definitive chemoradiation therapy with concurrent capecitabine receiving 45 Gy in 25 fx to the gastric mass and regional lymph nodes with a boost to the gross disease of 3.6 Gy in 2 fx (anticipated 9 Gy in 5 fx) using IMRT and IGRT. His boost treatment was ended early due to hospitalization for pneumonia. The total dose delivered was 48.6 Gy in 27 fx.

In the beginning of his treatment course, he tolerated the treatment well, but did report a lack of appetite and weight loss. On review of treatment imaging his lack of appetite and weight loss could have been attributed to dysmotility and inability to empty stomach. Patient was sent to the emergency room when he experienced a syncopal episode. Patient was diagnosed and treated for a urinary tract infection and pneumonia. However, two weeks later the patient and family decided to end chemoradiation early and pursue hospice care.

## Discussion

Even though gastric cancer is highly prevalent globally, early gastric cancer patients can have excellent prognosis with 90% survival rate at five years. However, over 70% of patients with gastric cancer have locally advanced gastric cancer at the time of diagnosis due to lack of symptoms [3]. For most patients, definitive treatment includes surgery with or without neoadjuvant chemotherapy or chemoradiation followed by possible adjuvant chemotherapy or chemoradiation [3-4]. Although, in the USA, surgical resection is not performed in greater than one-third of the patients with stage I-III gastric cancer due to comorbidities or anatomic unresectability [4].

Currently, no consensus on the preferred management for unresectable gastric cancer has been reached. Some potential approaches include palliative chemoradiation to improve gastric cancer patients’ overall quality of life [3-4]. Table 1 shows studies using definitive chemoradiation for gastric cancer.

**Table 1:** Comparison of Studies Performed on Definitive Chemoradiation in Gastric Cancer Patients ECOG-Eastern Cooperative Oncology Group PS-Performance Status T- Tumor N-Nodal

Study (First Author)	Number of Patients Receiving Chemoradiation	Chemoradiation Tumor and Nodal Stage	Performance Status	Median Radiation Dose	Chemotherapy	Median Follow Up Time	Median survival
Li et al. 2018 [4]	1,479	T1-32%, T2-39.9%, T3- 34.9 %, T4-29.6 %, Unknown-23.6 %, N0-34.8%, N1-31.4%, N2-36.9%, N3-31.0 %, Unknown-21.6%	Not reported	45 Gy	Not reported	11.8 months	12.3 months
Kaya et al. 2018 [5]	57	T1/T2-19.7 %, T3-66.2 %, T4-14.1%, N0- 45.1 %, N+- 54.9 %	Poor PS-19.7 %	45 Gy	Fluoropyrimidine alone or with a Platinum compound	21.6 months	26.4 months
Schein 1982 [6]	45	Not reported	PS 0-1=64 %, PS 2-3=36 %	50 Gy	5‐fluorouracil and Methyl-CCNU	48 months	9 months
Wydmański [7]	13	T1-T2 38.5 %,T3-61.5 %,N0 61.5 %,N1-N3 38.5 %	PS 0-46.1 %, PS 1-38.5%, PS 2-15.4 %	45 Gy	5‐fluorouracil.	30.1 months	17.1 months
Liu et al. 2017 [8]	36	T1-T2 :28 %, T3-58 %, T4-14 %, N0-28%, N1-42%, N2-25%, N3-5%	PS 0-36%, PS 1-45%, PS 2-19 %	50.4 Gy	Docetaxel	35.6 months	25.8 months
Anthony et al. 2020 (Current study)	3	T2-33.3 %, T4, 66.7 %,N0-66.7 %, N1-33.3%	ECOG PS 2-100 %	48.6-65 Gy	Capecitabine	3 months	10 months

Extrapolating from locally advanced esophageal carcinoma data, definitive chemoradiation may be an appropriate alternative in nonsurgical individuals [9,10]. However, there is a lack of data on definitive chemoradiation therapy in locally advanced gastric cancer patients with poor performance status and comorbidities. As presented in our cases 1 and 2, definitive chemoradiation was shown to be an effective alternative with reduction in the size of the known gastric cancer mass as observed on follow-up imaging. The median survival, which was defined as the amount of time from diagnosis to hospice care and/or death, was 10 months for our three cases. Furthermore, definitive chemoradiation was generally well tolerated in our patients and side effects were managed with medical treatment throughout the treatment regimen. We understand the small sample size of this case series and that larger prospective studies are indicated to better understand the role of definitive chemoradiation in locally advanced gastric cancer. Possible reasons that definitive chemoradiation has not commonly been used for gastric cancer in the past include difficulties in defining the target in the 2D and 3D eras, deformation, motion management, proximity of nearby organs at risk. Some of these problems may be overcome with MR linac and adaptive planning [11].

Li et al. published a National Cancer Database study of 4,795 comparing the effectiveness of chemotherapy to chemoradiation when surgery could not be performed in patients with non-metastatic adenocarcinoma gastric cancer. They reported that chemoradiation can improve survival in patients with unresected gastric cancer with a 24-month overall survival rate of 28.3% and median survival of 12.3 months, in comparison to chemotherapy which had an overall 24-month survival rate of 21.5% and median survival of 11.3 months [4]. Similar to the presentation of our three cases, their patient population who received chemoradiation had an older age at diagnosis and showed a slightly higher median survival than our reported 10 months [4]. 

Kim et al. studied adjuvant chemoradiation but retrospectively reviewed 39 patients with gastric cancer who underwent definitive chemoradiation therapy due to metastasis, poor performance status, and/or personal choice had a similar median overall survival of 10.1 month [6]. In contrast, other studies performed by Kaya et al., Wydmanski et al., and Liu et al. showed a higher median survival of 26.4 months, 17.1 months, and 25.8 months, respectively, in a patient population with a slightly higher performance status [5,7,8]. 

Given the lack of consensus on the use of definitive chemoradiation in locally advanced gastric cancer patients, our three presented cases show that definitive chemoradiation with dose escalation may be a viable alternative treatment option in gastric cancer patients with poor performance status and comorbidities. Larger studies are needed to establish the role of definitive chemoradiation therapy in the management of unresectable gastric cancer. 

## Conclusions

The use of definitive chemoradiation in locally advanced gastric cancer patients is not commonly utilized, however, our three presented cases show that definitive chemoradiation with dose escalation may be a viable alternative treatment option in gastric cancer patients with poor performance status and comorbidities. Larger studies are needed to establish the role of definitive chemoradiation therapy in the management of unresectable gastric cancer.
